# John Birtchnell, MD, DPM, FRCPsych, FBPsS

**DOI:** 10.1192/bjb.2023.1

**Published:** 2023-06

**Authors:** Sandra Birtchnell

Formerly Scientific Officer, Medical Research Council Social and Community Psychiatric Unit, Institute of Psychiatry, London, UK

**Figure:**
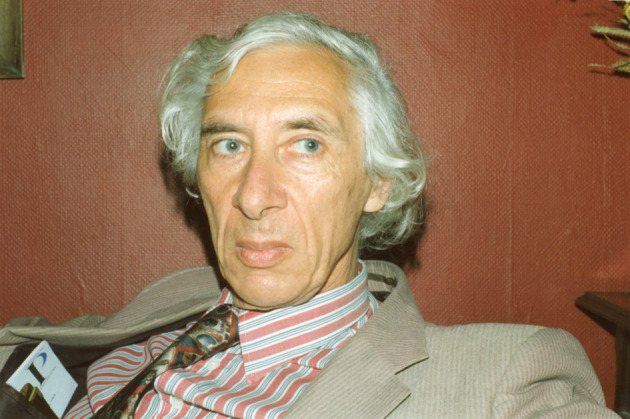

John Birtchnell, who died on 5 September 2022 at the age of 90, had a long and prolific career in psychiatric research. A common factor linking his areas of research interest was the definition and measurement of psychological dependence. It was out of this that he developed his general theory of relating, which he regarded as his most important contribution to knowledge.^1^ Questionnaires were constructed for measuring maladaptive forms of relating both between couples and between family members. The theory has proved useful in the assessment of personality disorder in a number of clinical settings. It has also been used in research in the evaluation of individual and couple psychotherapy.^2^ Having observed a strong association between marital quality and depression he began to develop measures of marital quality and carried out a collaborative study with marriage guidance counsellors and with the Tavistock Institute of Marital Studies in London. Out of this collaboration emerged numerous papers on the links between marital conflict, marital affection and depression in wives. Another strand to his research was a concern with the possible causes and management of attempted suicide, which led to links with the Samaritans.

Throughout his career John balanced his pursuit of statistically valid data from populations with the in-depth knowledge of individuals, couples and families gained through his clinical work as a psychotherapist. In over 100 published papers, topics included early loss and separation experiences, social class and mental illness, marital quality and depression, the causes and management of attempted suicide and maladaptive forms of relating. In later years John drew on evolutionary theory to theorise on brain function and was fascinated by aspects of memory even as his own was failing.

John was born in Aylesbury on 8 April 1932, the only child of Wilfred and Minnie Birtchnell. His father was an aircraft fitter. The family moved to Gloucester when he was five, his father taking a job in a nearby aircraft factory. John attended Sir Thomas Rich School. His schooling was interrupted when, at the age of 12, he fell from a tree, sustaining a compound fracture of his arm and nearly dying of septicaemia, being saved by the recent introduction of sulphonamides. A second medical emergency was the outcome of his quest for knowledge, when, at the age of 15, learning that six berries of deadly nightshade would be lethal, he thought it would be interesting to experience the effects of three.

Already confirmed in his wish to be a psychiatrist before he studied medicine at Edinburgh University, he did house jobs in psychiatry and neurosurgery, then senior house officer posts in Liverpool in neurology and psychiatry, obtaining the Diploma in Psychological Medicine in 1963. After clinical posts at Crichton Royal Hospital (Dumfries) and St John's Hospital (Aylesbury) he went to Aberdeen. His earliest research interests were in the possible short- and long-term consequences of parental death, the topic of his MD thesis, at Aberdeen University, where he subsequently trained full time in psychotherapy, obtaining the Aberdeen University Diploma in Psychotherapy.

For the remainder of his career he worked for the Medical Research Council, commencing as an MRC Clinical Research Fellow in the Department of Mental Health, Aberdeen University, where he worked on the Aberdeen Case Register, then moving in 1970 to the MRC Clinical Psychiatry Unit at Graylingwell Hospital, Chichester, under the directorship of Dr Peter Sainsbury. Here the focus was research on suicide and attempted suicide. On Dr Sainsbury's retirement in 1982, this unit was disbanded and John was relocated to the MRC Social Psychiatry Unit at the Institute of Psychiatry, London, where he worked for most of his career. After his retirement in 1998, he continued to work as an Attached Worker with the Section of Clinical Psychiatry at the Institute.

He was the editor of the *British Journal of Medical Psychology* (1989–1995) and it is a measure of his ability to work collaboratively across disciplines that, as well as being a Fellow of the Royal College of Psychiatrists, he was awarded the Fellowship of the British Psychological Society and Honorary Membership of the British Association of Art Therapists.

John was a keen amateur artist and developed an interest in art therapy while at Aberdeen. It was there that he made a film *A young man preoccupied with his nose*. He wrote papers on art therapy and contributed over a 20-year period to an annual residential art therapy school at Leeds Metropolitan University.

He married Rosemary (Niven) in 1961 and they divorced in 1969. He met his second wife, Sandra, in 1970, when she came on secondment to the MRC Unit in Chichester, the courtship ritual including writing two papers together. Following marriage in 1973, they had two sons. In later years, he developed Alzheimer's disease which had a chronic course. His illness spared him the grief of the loss of his younger son in 2020. He is survived by his wife, elder son and grandson.

## References

[1] Birtchnell J. How Humans Relate: A New Interpersonal Theory. Praeger, 1993.

[2] Birtchnell J. Relating in Psychotherapy: Application of a New Theory. Praeger, 1999.

